# Editorial: Circadian rhythm in adrenal endocrinology

**DOI:** 10.3389/fendo.2025.1620570

**Published:** 2025-05-14

**Authors:** Birgit Harbeck

**Affiliations:** III. Department of Medicine, University Medical Center Hamburg-Eppendorf, Hamburg, Germany

**Keywords:** circadian rhythm, circadian clock, circadian syndrome, adrenal endocrinology, postpartum depression, pheochromocytoma

Many physiological processes are under control of a master circadian clock located in the suprachiasmatic nucleus (SCN) of the hypothalamus, including endocrine functions. Besides regulating the body´s metabolism, this master clock synchronizes peripheral clocks in almost all cells in the body (1).

These circadian clocks are the key mechanisms that drive circadian rhythms. Circadian rhythms are physical, mental and behavioral changes that follow a 24-hour cycle, adjusting energy metabolism, inflammatory processes, cellular renewal as well as the interplay with the gut microbiota. Thus, biological circadian system is the major regulator of nearly every aspect of human health and metabolism and enables humans to adapt to changes in our environment.

The most well-known circadian rhythm is the sleep-wake cycle. Likewise cortisol, an essential steroid hormone, follows a circadian rhythm like many other physiological processes in the human body (e.g. blood pressure, heart rate (2).

Circadian disruption and concomitant sleep disturbances are present across a variety of psychiatric disorders (3), diabetes and metabolic syndrome as well as cardiovascular diseases (2, 4) and may be linked to malignant diseases (5).

Overall, four articles were published within this Research Topic ranging from basic science to clinical research. Otani et al. aimed to characterize the basic circadian clock properties of the adrenal zona glomerulosa (ZG) cells. They showed *in vitro* that ZG cells (rodent adrenal cells and its related H295R cells) possess genetically encoded, self-sustained and cell-autonomous peripheral circadian clock and are entrainable by Angiotensin type II. Using a type I Angiotensin II receptor inhibitor, caused alteration of the phase of the clock in human ZG cell line-H295R cells.

Another article investigated the association between circadian syndrome and chronic kidney disease (CKD) in an aging population (Xiong et al.). CKD is a progressive condition that affects >10% of the general population worldwide, especially older individuals (6). Circadian syndrome is proposed to comprise a risk cluster with reduced sleep duration, abdominal obesity, depression, hypertension, dyslipidemia and hyperglycemia. Therefore, in total 6355 participants with and without CKD were followed up for four years in a prospective cohort study. In brief, the authors found, that circadian syndrome is a risk factor for CKD and may serve as a predictor of CKD for early identification and intervention.

Chai et al. focused on the methodology problems in the investigation of postpartum depression (PPD) and hypothalamic-pituitary axis hormones. This Research Topic is of great importance as PPD has a prevalence of 10-15% worldwide (7) with half of them being unrecognized (8) although mother as well as offspring can suffer from severe complications. Thus, early diagnosis and treatment are very important. Abnormal function of the HPA axis was often found in patients with PPD (9). Taking this into account, evaluation of a biomarker should consider the compliance of patients during sampling, sampling type and time as well as costs. Methodological problems of studies published in the past decade were summarized by the authors. Although they found inconsistency of the studies with regard to conclusion, experimental design and methods, the combination of conventional behavioral assessments and regular hormonal work-up should be included in the regular screening for PPD in endangered patients according to the authors. Suggestions for reducing inconsistency in hormonal evaluations were made.

As disruption of the daily rhythms of cell metabolism may contribute to cancer development, the review of Tabebi et al. is very relevant. Cancer is one of the leading factors of death worldwide. Pheochromocytomas- catecholamine-secreting tumors of chromaffin cells most typically located in the adrenal glands- are characterized by endocrine disruption with non-circadian blood dysregulation (10), leading to stress and de-regulation of chromaffin cells (11). Around 60% of pheochromocytomas are due to known germline and somatic mutations (12), genetically linked to disrupted oxygen sensing and hypoxia signaling (13). The authors discussed in their review the molecular and physiological interplay between hypoxia signaling and the circadian clock in pheochromocytoma fostering endocrine disruption that leads to loss of circadian blood pressure variation. Potential tumor-specific therapeutic targets in the future were addressed.

In summary, this Research Topic provides new insights about the presence of Ang II-responsive molecular clock in rodent adrenal ZG cells and adrenocortical H295R cells and the relation between circadian syndrome and chronic kidney disease (CKD) in an aging population. Further *in vivo* studies will be required to investigate sex-derived differences in clock function in the ZG as well as the mechanisms by which circadian syndrome leads to CKD. Since the development of novel assessments for PPD are urgently needed, the combination of behavioral assessments and hormonal assessments may be a promising approach to promote the well-being of mothers and infants. The suggestions made by the authors for reducing inconsistencies in hormone examinations should be taken in mind. The review by Tabebi et al. improves the understanding of circadian disruption as a factor of progressive pheochromocytomas. Future research is warranted to explore potential therapeutic options.
